# Omega-3 fatty acids modulate cyclophosphamide induced markers of immunosuppression and oxidative stress in pigs

**DOI:** 10.1038/s41598-019-39458-x

**Published:** 2019-02-25

**Authors:** Sang In Lee, Kyung Soo Kang

**Affiliations:** 1 Department of Animal Resource and Science, Dankook University, Cheonan, Chungnam 330-714 Republic of Korea; 2 Bio Division, Medikinetics, Inc., Hansan-gil, Pyeongtaek-si, Gyeonggi-do, 17792 Republic of Korea

## Abstract

Immunosuppression directly correlates with economic benefits in livestock. Although omega-3, known as an energy source, is used as a pharmaceutical molecule, it remains unknown whether dietary supplementation with omega-3 can alleviate cyclophosphamide-induced immunosuppression in pigs. Omega-3 treatment increased the number of white blood cell, lymphocytes, and monocytes and decreased tumor necrosis factor (TNF)-α production under CTX challenge. In addition, we confirmed that omega-3 decreased the expression of nuclear factor (NF)-κB, TNF-α, interferon (IFN)-γ, and interleukin (IL)-8 in peripheral blood mononuclear cells. Additionally, omega-3 alleviated the activities of liver injury markers (alanine transaminase [ALT] and aspartate transaminase [AST]) and modulated oxidative stress markers (superoxide dismutase [SOD], malondialdehyde [MDA], and glutathione peroxidase [GPx]) in the blood serum after the CTX challenge. Based on these results, we suggest that omega-3 treatment modulates CTX-induced immunosuppression and oxidative stress in pigs. These results may have important implications in the development of new therapeutic approaches to improve immunosuppression, hepatic injury and dysfunction, and oxidative stress in pigs.

## Introduction

Immunosuppression induced by many factors such as infection and stress results in mortality, susceptibility to diseases, and growth retardation^1,2^. It is important to modulate immunosuppression because it directly correlates with economic benefits in livestock production. Cyclophosphamide (CTX) is one of the most commonly used drugs for immunosuppression induction^3,4^. CTX is converted by liver cytochrome P450 enzymes into its metabolite 4-hydroxycyclophosphamide, which has chemotherapeutic activity^5^. Despite the numerous adverse effects of CTX treatment such as pneumonitis, pulmonary fibrosis, bone marrow suppression, genotoxicity, and cardiotoxicity, it is frequently used in anticancer chemotherapy and for preconditioning the host for immunotherapy because of its immunosuppressive activity^6–10^.

Polyunsaturated fatty acids (PUFAs) as an energy source and membrane component, regulate the expression of genes involved in biological processes at the tissue, cellular, and molecular levels and could be used as pharmaceutical molecules^11,12^. Omega-3 fatty acids, eicosapentaenoic acid (EPA), and docosahexaenoic acid (DPA) are major dietary PUFAs derived from fish oil^13^. Numerous previous studies have demonstrated that omega-3 was used as pharmaceutical drug for inflammatory disease and genotoxicity, and oxidative stress^13,14^. However, it remains unknown whether omega-3 treatment could alleviate the immunosuppressive effects of CTX on inflammation and oxidative stress in pigs.

The choice of an animal model is important in investigating the pathogenesis, and virulence, immunology, as well as diagnostic criteria^15^. The advantages of rodents such as mice and rats as biomedical models include the ease of producing genetically modified species, which facilitates experimental management. However, in some cases, mice are not appropriate as animal models for studying human nutrition and metabolism because of morphological and physiological differences in the organs of humans and rodents^16^. Pigs, as a large animal model, are acceptable for studying human nutrition and metabolism because of they have more similarities to humans in both genetic and disease characteristics than rodent models do^17,18^. Therefore, in the present study, we used a miniature pig model to evaluate the effects of feeding omega-3 fatty acids on CTX-induced immunosuppressed pigs.

## Results

### Effects of omega-3s on immune cells and tumor necrosis factor (TNF)-α production after CTX challenge

**Figure 1:**
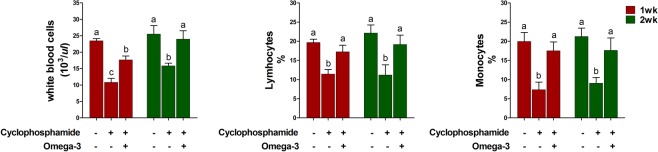
Effects of omega-3 on number of immune cells including white blood cells (**a**), lymphocytes (**b**), and monocytes (**c**) in blood 1 and 2 weeks after the CTX challenge. Miniature pigs were randomly allocated into three groups: (T1) control diet + saline challenge; (T2) control diet plus CTX challenge; and (T3) control diet with 0.5% omega-3 plus CTX challenge. Error bars indicate standard error of triplicate analyses. Lowercase letters (**a**–**c**) indicate significant differences (P < 0.05) between treatments based on Duncan’s multiple range test.

To determine whether omega-3 treatment affects the immune reaction after a CTX challenge, we counted the numbers of immune cells such as white blood cells (WBCs), lymphocytes, and monocytes in the blood after the CTX challenge. The CTX challenge decreased the numbers of WBCs, lymphocytes, and monocytes at 1 and 2 weeks compared to those in the controls (Fig. 1). However, omega-3 treatment increased the numbers of WBCs, lymphocytes, and monocytes at 1 and 2 weeks after the CTX challenge.

**Figure 2:**
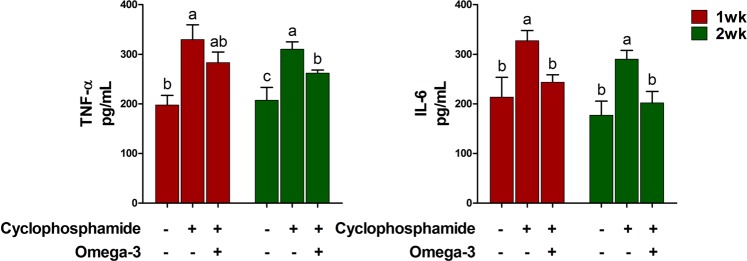
Effects of omega-3 on the production of the inflammatory cytokine TNF-α and IL-6 in serum 1 and 2 weeks after CTX challenge. Miniature pigs were randomly allocated into three groups: (T1) control diet + saline challenge; (T2) control diet plus CTX challenge; and (T3) control diet with 0.5% omega-3 plus CTX challenge. TNF-α was determined using ELISA (n = 5). Error bars indicate standard error of triplicate analyses. Lowercase letters (**a**–**c**) indicate significant differences (P < 0.05) between treatments based on Duncan’s multiple range test.

The TNF-α and interleukin (IL)-6 production was increased by CTX challenge compared to that in the controls at 1 and 2 weeks (Fig. 2). Under the CTX challenge, the levels of TNF-α and IL-6 were decreased by omega-3 treatment at 1 and 2 weeks.

### Effects of omega-3s on genes related to inflammatory cytokines in peripheral blood mononuclear cells after CTX challenge

**Figure 3:**
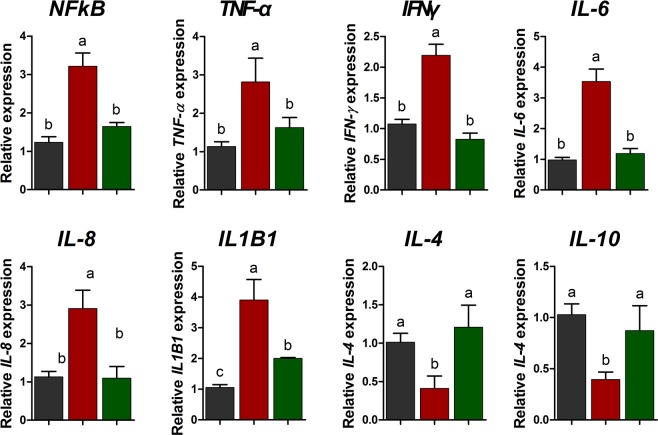
Quantitative expression of genes encoding NF-κB and inflammatory cytokines (*NFkB*, *TNF-α*, *IFN-γ*, *IL-6*, *IL-8*, *IL-1β1*, *IL-4*, and *IL-10*) in peripheral blood mononuclear cells after CTX challenge. The qRT-PCR data were normalized relative to expression of *GAPDH* as an endogenous control gene and calculated using the 2^−ΔΔCt^ method (n = 5). Error bars indicate standard error of triplicate analyses. Lowercase letters (**a**–**c**) indicate significant differences (P < 0.05) between treatments based on Duncan’s multiple range test. CON, control diet plus saline challenge (black bar); CTX, control diet plus CTX challenge (red bar); and CTX plus omega-3, control diet with 0.5% omega-3s plus CTX challenge (green bar).

We next examined whether omega-3 plays a role in modulating the production of cytokines through the nuclear factor (NF)-κB-mediated signaling pathway, which leads to the inflammatory gene expression in PBMCs (Fig. 3). The CTX challenge increased the expression of the genes encoding *NF-κB*, *TNF-α*, interferon-γ (*IFN-γ*), *IL-8*, and *IL-1β1* and decreased the expression of those encoding *IL-4* and *IL-10* compared with the control. However, omega-3 treatment decreased the expression of the genes encoding *NF-κB*, *TNF-α*, *IFN-γ*, *IL-6*, *IL-8*, and *IL-1β1* and increased the expression of those encoding *IL-4* and *IL-10* after the CTX challenge.

### Effects of omega-3s on alanine transaminase (ALT) and aspartate transaminase (AST) as liver function parameters after CTX challenge

**Figure 4:**
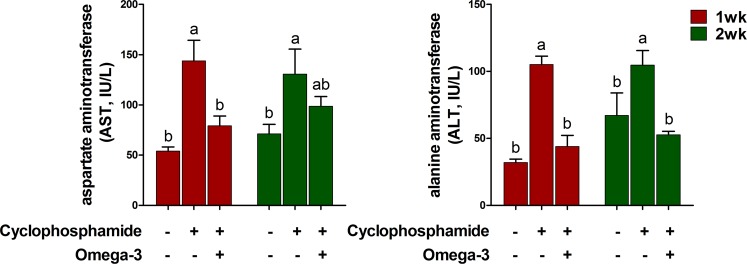
Effects of omega-3s on ALT and AST levels in the blood serum at 1 and 2 weeks after the CTX challenge. Miniature pigs were randomly allocated into three groups: (T1) control diet plus saline challenge; (T2) control diet plus CTX challenge; and (T3) control diet with 0.5% omega-3 plus CTX challenge. ALT and AST were determined by ELISAs (n = 5). Error bars indicate standard error of triplicate analyses. Lowercase letters (**a**,**b**) indicate significant differences (P < 0.05) between treatments based on Duncan’s multiple range test.

To determine whether omega-3 treatment affects the parameters of liver function after the CTX challenge, we examined the activities of AST and ALT in the blood serum 1 and 2 weeks after the CTX challenge. The CTX challenge increased the activities of AST and ALT at 1 and 2 weeks compared to those in the control (Fig. 4). However, omega-3 treatment decreased the activity of AST at 2 weeks and that of ALT at 1 and 2 weeks after the CTX challenge.

### Effects of omega-3s on oxidative stress markers after CTX challenge

**Figure 5:**
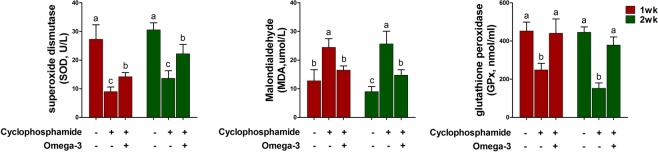
Effects of omega-3 on SOD (**a**), MDA (**b**), and GPx (**c**) levels in the blood serum at 1 and 2 weeks after the CTX challenge. Miniature pigs were randomly allocated into three groups: (T1) control diet plus saline challenge; (T2) control diet plus CTX challenge; and (T3) control diet with 0.5% omega-3 plus CTX challenge. SOD, MDA, and GPx were determined by ELISAs (n = 5). Error bars indicate standard error of triplicate analyses. Lowercase letters (**a**–**c**) indicate significant differences (P < 0.05) between treatments based on Duncan’s multiple range test.

To determine whether omega-3 treatment affects the levels of oxidative stress markers after the CTX challenge, we examined the activities of superoxide dismutase (SOD) and glutathione peroxidase (GPx) and the level of malondialdehyde (MDA) in the blood serum at 1 and 2 weeks after the CTX challenge. The CTX challenge decreased the activities of SOD and GPx and increased the level of MDA at 1 and 2 weeks compared to those in the control (Fig. 5). However, omega-3 treatment increased the activities of SOD and GPx and decreased the level of MDA 3 and 4 weeks after the CTX challenge.

**Figure 6:**
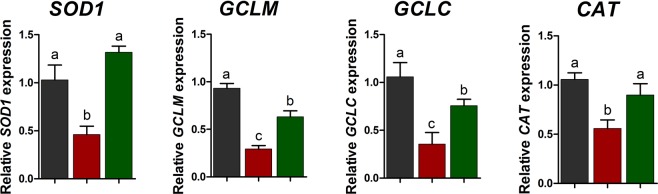
Quantitative expression of the genes encoding antioxidant enzymes (*SOD1*, *GCLM*, *GCLC*, and *CAT*) in peripheral blood mononuclear cells after the CTX challenge. The qRT-PCR data were normalized relative to the expression of *GAPDH* as an endogenous control gene and calculated using the 2^−ΔΔCt^ method (n = 5). Error bars indicate standard error of triplicate analyses. Lowercase letters (**a**–**c**) indicate significant differences (P < 0.05) between treatments based on Duncan’s multiple range test. CON, control diet plu saline challenge (black bar); CTX, control diet plus CTX challenge (red bar); and CTX plus omega-3, control diet with 0.5% omega-3s plus CTX challenge (green bar).

We next examined whether omega-3 affects the expression of antioxidant enzymes in PBMCs (Fig. 6). The CTX challenge decreased the expression of SOD1, CGLM, CGLC, and catalase (CAT) compared to the control. However, omega-3 treatment increased the expression of SOD1, CGLM, CGLC, and CAT after the CTX challenge.

## Discussion

CTX is widely used as a potent immunosuppressive agent for organ transplantation and treatment of various autoimmune disorders^1^. In livestock, immunosuppression reduces the growth performance including the feed intake, body weight (BW) gain, and feed conversion ratio, and increases oxidative stress, which can negatively impact economic benefits^1,19^. Therefore, investigating the mechanism of immunosuppression is very important for the improvement of growth performance in livestock. In many previous reports, omega-3 fatty acids have shown beneficial effects on oxidative stress and inflammation in animals^20–23^. In the present study, we examined whether omega-3 supplementation moderates the immune function and oxidative stress in CTX-challenged miniature pigs. It is well documented that compared to other non-rodent species, miniature pigs share many anatomical and physiological similarities with humans^24^. Furthermore, miniature pigs have the advantages of fewer ethical issues pertaining to the use of animals in biomedical research, and they have been shown to be sensitive to a wide variety of drugs and chemicals^24–26^. In the present study, we used the MK strain of miniature pigs as an animal model system to investigate the effects of feeding omega-3 on liver injury, oxidative stress, and cytokine production.

The present study showed that omega-3 treatment increased the immune cell numbers such as WBCs, lymphocytes, and monocytes and decreased TNF-α and IL-6 production in the blood serum following CTX challenge. The present results are consistent with data from several other studies, which showed that omega-3 treatment decreased the TNF-α secretion^27,28^. It is widely accepted that omega-3 PUFAs have immunomodulatory effects, based on the data from human clinical and epidemiological studies, as well as on those obtained in murine models^13,29^. The immune system consists of factors that mediate innate and adaptive immunity, representing the first line of defense against invading pathogens and leading to immunological memory, respectively^30^. NF-κB is the key regulator of both innate and adaptive immunity in the immune system. Toll-like receptors, which are transmembrane proteins expressed in immune cells, including macrophages, play a critical role in immunostimulatory molecules, activate NF-κB^31,32^. The present study investigated the effects of omega-3 treatment on the expression of genes related to pro- and anti-inflammatory cytokines in PBMCs and found it treatment decreased the levels of pro-inflammatory cytokines and increased those of anti-inflammatory cytokines in the serum in porcine PBMCs after the CTX challenge.

The present study revealed that omega-3 treatment improved liver function parameters, as indicated by decreased levels of ALT and AST in the blood serum under the CTX challenge, which induces liver injury. It is widely accepted that CTX treatment causes significant hepatotoxic effects, which has been confirmed by increased activities of ALT, AST, and ALPand serum enzymes indicating cellular damage and a loss of functional integrity of the cell membrane in the liver^33,34^. In addition, the present study revealed that omega-3 treatment modulated the SOD, GPx, and MDA activities in the serum and increased the expression of antioxidant enzymes (SOD1, CGLM, CGLC, and CAT) in PBMCs after CTX challenge. These results are consistent with the data from previous studies, which showed protective effects of omega-3s against genotoxicity and oxidative stress in CTX-induced mice^14,35^. In a previous report, mice in the omega-3 dietary group exhibited significantly higher liver CAT, SOD, and GPx than those administered CTX without omega-3s, which suggested that omega-3s could provide a beneficial effect in hepatic tissues subjected to CTX-induced oxidative stress by regulating activities of antioxidant enzymes^35^. In other previous studies, activities of SOD and CAT and the extent of lipid peroxidation statistically significantly increased in liver cells of the mice exposed to omega-3s compared to the levels observed in the CTX-induced mice not receiving omega-3s^14^. Based on the results, we suggested that the chemopreventive actions of omega-3s might be partially attributed to elevation of the levels of enzymatic antioxidants. Thus, omega-3 fatty acids may be a potential antigenotoxic, antioxidant, and chemopreventive agent and could be used as an adjuvant in chemotherapy.

In conclusion, omega-3 treatment increased the numbers of WBCs, lymphocytes, and monocytes and decreased the TNF-α production after CTX challenge in pigs. In addition, omega-3 treatment decreased the expression of *NF-κB*, *TNF-α*, *IFN-γ*, and *IL-8* genes and increased that *IL-10* in PBMCs. AST and ALT activities, which are liver function parameters, were decreased by omega-3 treatment after the CTX challenge. Moreover, after the CTX challenge, omega-3 treatment enhanced the activity of SOD and decreased the level of MDA, which are oxidative stress markers in the blood serum. Based on these results, we suggest that omega-3 treatment alleviates the CTX-induced immunosuppression, in pigs. These results may have important implications in the development of new therapeutic approaches to ameliorate immunosuppression in pigs.

## Material and Methods

The animal care and experimental protocols of the present study were approved by the Animal Care, and Use Committee of Dankook University and all methods were performed in accordance with the relevant guidelines and regulations.

### Experimental design, feeding, and CTX challenge

Fifteen 100-day-old male miniature pigs [MK strain, (Duroc × Yorkshire) × (Pot Valley × Berkshire) × Yucatan] with an average initial BW of 21.73 ± 0.43 kg were used to evaluate the effects of feeding omega-3 after a CTX challenge in a 28-day (4-week) feeding trial. Each pig was kept in an individual pen measuring 1.8 m × 1.8 m and housed in an environmentally controlled nursery facility with slatted plastic flooring and a mechanical ventilation system. The temperature and humidity of the room were maintained at approximately 27 °C, and 60%, respectively. Ventilation was provided by a mechanical system with automatic adjustments to provide 12 h artificial light per day. Each pen was equipped with a one-sided, stainless steel self-feeder and a nipple drinker, which allowed *ad libitum* access to feed and water. Experimental treatments were as follows: (T1) control diet plus saline challenge; (T2) control diet plus CTX challenge; and (T3) control diet with 0.5% omega-3 plus CTX challenge. The control diet was based on corn and soybean meal.

For the challenge assay, all pigs from each dietary treatment group were injected intraperitoneally with CTX or a saline solution. CTX (Sigma Aldrich) was diluted in a sterile saline solution and injected at 0.01% (50 mg/kg) of BW after a 14-day feeding. The dose of CTX used was based on the results of a previous study^19^. No vaccines or antibiotics were used in this experiment.

### Omega-3 fatty acids

The omega-3 fatty acid was kindly provided by a commercial company (Morningbio Co., Ltd, Cheonan, Korea). It was extracted from linseed oil using drying method according to a previous report^36^. The omega-3 fatty acids contain 55.75% alpha-linolenic acid, 13.09% EPA, 15.16% DHA, 7.24% palmitic acid, and 5.23% oleic acid.

### Blood collection and biochemical analysis

At the end of the experiment, blood samples were collected and analyzed according to our standard protocol^37^. Briefly, blood samples were collected from all pigs via jugular venipuncture 1 and 2 weeks after the challenge into a non-heparinized K_3_EDTA vacuum tube (Becton Dickinson Vacutainer systems) to obtain serum and whole blood. Leukocyte, lymphocyte, and monocyte counts were determined using an automatic blood analyzer (ADVIA 120; Bayer).

The whole blood samples were subsequently centrifuged at 3,000 × *g* for 15 min at 4 °C, and the serum was harvested. Then, the samples were frozen and stored at −20 °C until further analysis. The level of serum TNF-α was determined using an enzyme-linked immunosorbent assay (ELISA) kit (R&D Systems, USA). The activities of ALT, AST (both Sigma-Aldrich, USA), SOD (Cohesion Biosciences, USA), and GPx (Cayman Chemical, USA), as well as the level of MDA (Abcam, UK) in the serum were assayed using commercial kits.

### Peripheral blood mononuclear cell preparation

For PBMC isolation, 15 blood samples were collected into a K_3_EDTA vacuum tube 2 weeks after the challenge. The PBMCs were prepared according to a previous study^38^. Briefly, the collected blood samples were diluted with an equal volume of a balanced salt solution, and PBMCs were immediately isolated using Histopaque density gradient centrifugation according to the manufacturer’s instruction (Sigma–Aldrich). The diluted blood samples were mixed with half the volume of a Histopaque solution and then centrifuged at 400 × *g* for 35 min at room temperature. PBMCs were carefully aspirated from the Histopaque solution plasma interface.

### Real-time reverse transcription-quantitative polymerase chain reaction (RT-qPCR**)**

RNA was isolated using the TRIzol reagent (Invitrogen). For real-time reverse transcription- quantitative polymerase chain reaction (RT-qPCR), total RNA (100 μg) was used for complementary DNA synthesis using the Maxima first-strand cDNA synthesis kit (Life Technologies). The primers for RT-qPCR of each gene transcript were designed using the Primer3 program (http://frodo.wi.mit.edu/) (Table 1). RT-qPCR analysis was performed using a 7500 Fast real-time PCR system (Applied Biosystems). The RT-qPCR conditions were as follows: 94 °C for 3 min, followed by 40 cycles at 94 °C for 30 s, 59–61 °C for 30 s, and 72 °C for 30 s. Melting curve profiles were analyzed for the amplicons. The RT-qPCR data were normalized relative to the expression of glyceraldehyde 3-phosphate dehydrogenase (*GAPDH*) as an endogenous control gene and calculated using the 2^−ΔΔCt^ method, where ΔΔCt (cycle threshold) = ΔCt (treated) − ΔCt (control) and ΔCt = Ct of the target gene − Ct of *GAPDH* (treated or control, respectively)^39^.

**Table 1:** List of primers.

Gene symbol	Description	Accession No.	Forward (5′->3′)	Reverse (5′->3′)
*TNF-α*	Tumor necrosis factor alpha	NM_214022	TCTCCTTCCTCCTGGTCGCA	TCCCTCGGCTTTGACATTGG
*IL1B1*	Interleukin-1 beta1	NM_214055	CCGAAGCTGACAGAAGGGGA	AGTGGATGGGGCCTGAGGAT
*IL-8*	Interleukin-8	NM_213867	GGCTGTTGCCTTCTTGGCAG	TTTGGGGTGGAAAGGTGTGG
*IFN-γ*	Interferon gamma	NM_213948	GGCCATTCAAAGGAGCATGG	GATGGCTTTGCGCTGGATCT
*NF-κB*	Nuclear factor of kappa light polypeptide gene enhancer in B-cells 1	NM_001048232	GACAACATCTCCTTGGCGGG	TCTGCTCCTGCTGCTTTGAGG
*IL-4*	Interleukin-4	NM_214123	TCCACGGACACAAGTGCGAC	TGTTTGCCATGCTGCTCAGG
*IL-6*	Interleukin-6	NM_214399	AGCCCACCAGGAACGAAAGA	AGCCATCACCAGAAGCAGCC
*IL-10*	Interleukin-10	NM_214041	CATCCACTTCCCAACCAGCC	CTCCCCATCACTCTCTGCCTTC
*SOD1*	superoxide dismutase 1	NM_001190422	GTACCAGTGCAGGTCCTCAC	TTTGCCAGCAGTCACATTGC
*GCLM*	glutamate-cysteine ligase modifier subunit	XM_001926378	TTGGAGCAGCTGTACCAGTG	GAGCTTCCTGGAAACTCGCT
*GCLC*	glutamate-cysteine ligase catalytic subunit	XM_003482164	GTCCAGTTGGTCCTGTCTGG	CGGGAGTCCCTTCGATCATG
*CAT*	catalase	NM_214301	ACACAGGCACATGAACGGAT	GTCCCGGATGCCATAGTCAG
*GAPDH*	Glyceraldehyde-3-phosphate dehydrogenase	NM_001206359	AATGGGGTGATGCTGGTGCT	GGCAGAAGGGGCAGAGATGA

### Statistical analysis

The data were statistically analyzed using an analysis of variance (ANOVA) using the general linear model (GLM) procedure of statistical analysis software (SAS) program (SAS Institute, NC, USA) with a completely randomized design. The data are presented as the means standard error of the means (SEM). To determine the significant difference between treatments in the immune cells, TNF-α production, inflammatory cytokines-related gene expression, liver function parameters, and oxidative stress markers were analyzed using the GLM in SAS. Duncan’s multiple range test was used as post-hoc test was used to analyze the differences between means and a *p* < 0.05 was considered statistically significant.

## References

[1] El-Abasy M (2004). Preventive and therapeutic effects of sugar cane extract on cyclophosphamide-induced immunosuppression in chickens. International immunopharmacology.

[2] Tayade C, Jaiswal TN, Mishra SC, Koti M (2006). L-arginine stimulates immune response in chickens immunized with intermediate plus strain of infectious bursal disease vaccine. Vaccine.

[3] Shirani K (2015). Phytotrapy of cyclophosphamide-induced immunosuppression. Environmental toxicology and pharmacology.

[4] Kumar VP, Venkatesh YP (2016). Alleviation of cyclophosphamide-induced immunosuppression in Wistar rats by onion lectin (Allium cepa agglutinin). Journal of ethnopharmacology.

[5] Huttunen KM, Raunio H, Rautio J (2011). Prodrugs–from serendipity to rational design. Pharmacol Rev.

[6] Ochoa R, Bejarano PA, Gluck S, Montero AJ (2012). Pneumonitis and pulmonary fibrosis in a patient receiving adjuvant docetaxel and cyclophosphamide for stage 3 breast cancer: a case report and literature review. Journal of medical case reports.

[7] Shepherd JD (1991). Mesna versus hyperhydration for the prevention of cyclophosphamide-induced hemorrhagic cystitis in bone marrow transplantation. Journal of clinical oncology: official journal of the American Society of Clinical Oncology.

[8] Paul R, Kulkarni P, Ganesh N (2011). Avocado fruit (Persea americana Mill) exhibits chemo-protective potentiality against cyclophosphamide induced genotoxicity in human lymphocyte culture. J Exp Ther Oncol.

[9] Ozkan HA, Bal C, Gulbas Z (2014). Assessment and comparison of acute cardiac toxicity during high-dose cyclophosphamide and high-dose etoposide stem cell mobilization regimens with N-terminal pro-B-type natriuretic peptide. Transfus Apher Sci.

[10] Salem ML (2010). Cyclophosphamide induces dynamic alterations in the host microenvironments resulting in a Flt3 ligand-dependent expansion of dendritic cells. J Immunol.

[11] Ross JA, Moses AG, Fearon KC (1999). The anti-catabolic effects of n-3 fatty acids. Current opinion in clinical nutrition and metabolic care.

[12] Simopoulos AP (2002). Omega-3 fatty acids in inflammation and autoimmune diseases. Journal of the American College of Nutrition.

[13] Calder PC (2006). N-3 polyunsaturated fatty acids, inflammation, and inflammatory diseases. The American journal of clinical nutrition.

[14] Li M (2011). Protective effects of eicosapentaenoic acid on genotoxicity and oxidative stress of cyclophosphamide in mice. Environmental toxicology.

[15] Capilla J, Clemons KV, Stevens DA (2007). Animal models: an important tool in mycology. Medical mycology.

[16] Guo W, Yang SM (2015). Advantages of a miniature pig model in research on human hereditary hearing loss. Journal of otology.

[17] Bassols A (2014). The pig as an animal model for human pathologies: A proteomics perspective. Proteomics. Clinical applications.

[18] Smith DW, Harding GE (1977). Animal model of human disease. Pulmonary tuberculosis. Animal model: Experimental airborne tuberculosis in the guinea pig. The American journal of pathology.

[19] Han J (2009). Dietary L-arginine supplementation alleviates immunosuppression induced by cyclophosphamide in weaned pigs. Amino acids.

[20] Kajarabille N (2016). Omega-3 LCPUFA supplement: a nutritional strategy to prevent maternal and neonatal oxidative stress. Maternal & child nutrition.

[21] Amador-Licona N (2016). Omega 3 Fatty Acids Supplementation and Oxidative Stress in HIV-Seropositive Patients. A Clinical Trial. PloS one.

[22] Abdel-Dayem MA (2014). Valproate-induced liver injury: modulation by the omega-3 fatty acid DHA proposes a novel anticonvulsant regimen. Drugs in R&D.

[23] Nordgren TM (2014). The omega-3 fatty acid docosahexaenoic acid attenuates organic dust-induced airway inflammation. Nutrients.

[24] Svendsen O (2006). The minipig in toxicology. Experimental and toxicologic pathology: official journal of the Gesellschaft fur Toxikologische Pathologie.

[25] Jordan HL (2014). Nontraditional applications in clinical pathology. Toxicologic pathology.

[26] Ganderup NC, Harvey W, Mortensen JT, Harrouk W (2012). The minipig as nonrodent species in toxicology–where are we now?. International journal of toxicology.

[27] Chang HR (1992). Dietary supplementation with fish oil enhances *in vivo* synthesis of tumor necrosis factor. Immunology letters.

[28] Blok WL (1992). Dietary fish-oil supplementation in experimental gram-negative infection and in cerebral malaria in mice. The Journal of infectious diseases.

[29] Cathcart ES, Mortensen RF, Leslie CA, Conte JM, Gonnerman WA (1987). A fish oil diet inhibits amyloid P component (AP) acute phase responses in arthritis susceptible mice. J Immunol.

[30] Carpenter S, O’Neill LAJ (2007). How important are Toll-like receptors for antimicrobial responses?. Cellular microbiology.

[31] Lee J (2009). Nuclear factor kappaB (NF-kappaB) activation primes cells to a pro-inflammatory polarized response to a Toll-like receptor 7 (TLR7) agonist. The Biochemical journal.

[32] Zhang G, Ghosh S (2001). Toll-like receptor-mediated NF-kappaB activation: a phylogenetically conserved paradigm in innate immunity. The Journal of clinical investigation.

[33] Germoush MO, Mahmoud AM (2014). Berberine mitigates cyclophosphamide-induced hepatotoxicity by modulating antioxidant status and inflammatory cytokines. Journal of cancer research and clinical oncology.

[34] Kumar G, Banu GS, Kannan V, Pandian MR (2005). Antihepatotoxic effect of beta-carotene on paracetamol induced hepatic damage in rats. Indian journal of experimental biology.

[35] Bhattacharya A (2003). Effect of dietary n-3 and n-6 oils with and without food restriction on activity of antioxidant enzymes and lipid peroxidation in livers of cyclophosphamide treated autoimmune-prone NZB/W female mice. Journal of the American College of Nutrition.

[36] Watanabe Y, Fang X, Minemoto Y, Adachi S, Matsuno R (2002). Suppressive effect of saturated acyl L-ascorbate on the oxidation of linoleic acid encapsulated with maltodextrin or gum arabic by spray-drying. Journal of agricultural and food chemistry.

[37] Li J, Kim IH (2013). Effects of levan-type fructan supplementation on growth performance, digestibility, blood profile, fecal microbiota, and immune responses after lipopolysaccharide challenge in growing pigs. Journal of Animal Science.

[38] Lee SI, Kim HS, Koo JM, Kim IH (2016). Lactobacillus acidophilus modulates inflammatory activity by regulating the TLR4 and NF-kappaB expression in porcine peripheral blood mononuclear cells after lipopolysaccharide challenge. The British journal of nutrition.

[39] Livak KJ, Schmittgen TD (2001). Analysis of relative gene expression data using real-time quantitative PCR and the 2(-Delta Delta C(T)) Method. Methods.

